# Examining Disparities in Ownership and Use of Digital Health Technology Between Rural and Urban Adults in the US: An Analysis of the 2019 Health Information National Trends Survey

**DOI:** 10.7759/cureus.38417

**Published:** 2023-05-02

**Authors:** Emeka Okobi, Aisha O Adigun, Oyintoun-emi Ozobokeme, Omotola Emmanuel, Precious A Akinsanya, Omolola Okunromade, Okelue E Okobi, Henry O Aiwuyo, Anthony I Dick, Rasheedat A Sadiq-Onilenla, Foluke A Ogunlana

**Affiliations:** 1 Dentistry, Ahmadu Bello University Teaching Hospital Zaria, Abuja, NGA; 2 Division of Infectious Diseases, University of Louisville, Louisville, USA; 3 Medicine, Central Michigan University College of Medicine, Mount Pleasant, USA; 4 Clinical Documentation, Emory Hospital, Georgia, USA; 5 Oncology, Holy Name Medical Center, Teaneck, USA; 6 Public Health/Community Health Behavior & Education, Jiann-Ping Hsu College of Public Health, Georgia Southern University, Greater Savannah Area, USA; 7 Family Medicine, Medficient Health Systems, Laurel, USA; 8 Family Medicine, Lakeside Medical Center, Belle Glade, USA; 9 Internal Medicine, Brookdale University Hospital Medical Center, Brooklyn, USA; 10 Public Health, Chicago State University, Chicago, USA; 11 Health Sciences, Franklin University, Ohio, USA; 12 Family Medicine/General Practice, National Health Service (NHS) Foundation Trust Derbyshire, Derby, GBR

**Keywords:** information technology, mobile health, digital health tools, disparities, rural health

## Abstract

Background: Although research shows that digital health tools (DHT) are increasingly integrated with healthcare in the United States, very few studies have investigated the rural-urban differences in DHT adoption at the national level. Individuals in rural communities experience disproportionately greater rates of chronic diseases and face unique challenges in accessing health care. Studies have shown that digital technology can improve access and support rural health by overcoming geographic barriers to care.

Objective: To evaluate the rates of ownership and preferences for utilization of DHT as a measure of interest among rural adults compared to their urban counterparts in the United States using a National Inpatient Survey.

Methods: Data was drawn from the 2019 (n= 5438) iteration of the Health Information National Trends Survey (HINTS 5 cycle 3). Chi-square tests and weighted multivariable logistic regressions were conducted to examine rural-urban differences regarding ownership, usage, and use of digital health tools to interact with health care systems while adjusting for health-related characteristics and sociodemographic factors.

Results: The ownership rates of digital health technology (DHT) devices, including tablets, smart phones, health apps, and wearable devices, were comparable between rural and urban residents. For tablets, the ownership rates were 54.52% among rural residents and 60.24% among urban residents, with an adjusted odds ratio (OR) of 0.87 (95% confidence interval {CI}: 0.61, 1.24). The ownership rates of health apps were 51.41% and 53.35% among rural and urban residents, respectively, with an adjusted OR of 0.93 (95% CI: 0.62, 1.42). For smartphones, the ownership rates were 81.64% among rural residents and 84.10% among urban residents, with an adjusted OR of 0.81 (95% CI: 0.59, 1.11). Additionally, rural residents were equally likely to use DHT in managing their healthcare needs. Both groups were equally likely to have reported their smart device as helpful in discussions with their healthcare providers (OR 0.90; 95% CI 63 - 1.30; p = 0.572). Similarly, there were similar odds of reporting that DHT had helped them to track progress on a health-related goal (e.g., quitting smoking, losing weight, or increasing physical activity) (OR 1.17; 95% CI 0.75 - 1.83; p = 0.491), and to make medical decisions (OR 1.05; 95% CI 0.70 - 1.59; p = 0.797). However, they had lower rates of internet access and were less likely to use DHT for communicating with their healthcare providers.

Conclusion: We found that rural residents are equally likely as urban residents to own and use DHT to manage their health. However, they were less likely to communicate with their health providers using DHT. With increasing use of DHT in healthcare, future research that targets reasons for geographical digital access disparities is warranted.

## Introduction

Despite significant advances in health care policies and implementation, individuals residing in rural areas in the United States continue to experience poorer health outcomes than their urban counterparts [1]. A recent review suggests that those living in rural communities face challenges related to lack of availability of primary and specialist services, geographic barriers to accessing healthcare, longer travel and wait times, as well as, shortage of health service providers [2]. These barriers are further accentuated by the staggering disparities in socioeconomic conditions and health behaviors that exist between rural and urban populations [3]. As a result, rural Americans experience greater morbidity and mortality [4]. Recently, increasing attention has been drawn to this ongoing public health problem and research, as well as effort by governmental and non-governmental organization to narrow this widening gap, are ongoing. Digital health technology has emerged as one strategy to provide and augment care for rural residents [5].

Digital health technology refers to the use of digital tools and platforms to improve healthcare outcomes [5-11]. It encompasses a wide range of technologies, including telehealth, mobile apps, and wearables [1-3,5-11]. Digital health technology has the potential to improve the accuracy of diagnosis and treatment, enhance the delivery of healthcare, and increase access to care, especially in underserved rural areas [6-8]. By using telehealth, patients in rural areas can receive medical consultations and diagnoses from specialists located in urban areas, improving access to care and reducing travel costs [5-11]. Digital health technology can also help reduce healthcare disparities by improving the efficiency and accuracy of healthcare delivery [5]. For example, wearables and mobile apps can help patients monitor their health and manage chronic conditions, reducing hospital readmissions and improving outcomes [4-11]. However, the deployment of digital health technology requires a supportive regulatory environment, a culture of innovation, leadership commitment to clinical quality and public health, and a foundational data governance framework. Without these key elements, digital health technology may not be used effectively or may exacerbate existing healthcare disparities [4-6].

Digital health technology has revolutionized the way healthcare is delivered and managed, but its adoption and utilization vary widely between rural and urban populations in the United States. This disparity in the use of digital health technology is a significant challenge to achieving equitable access to healthcare services. Although early evidence indicates that technology based interventions such as telehealth are feasible and may result in improved health care access [6-8], prior research suggests that geographical disparities in digital engagement exist and that rural residents continue to lag behind their urban counterparts with respect to utilization of health information technology [9-11]. For example, in their large national study, Greenberg et al. utilized data from 2003 to 2014 and found that rural participants were less likely than urban participants to utilize technology to engage with the health care system and also less likely to endorse having access to the internet [10].

Similar findings were observed in studies conducted at the state level by Haggstrom et al. and Whitacre et al. In a more recent study by Krakow et al., it was observed that rural residents were less likely to access their online medical records and were also less likely to receive provider encouragement to use their online records. While these studies provide earlier evidence of rural-urban disparities, they were conducted some time ago and some studies were limited to state-level data and thus were not nationally representative. The coronavirus disease 2019 (COVID-19) pandemic ushered and facilitated a rapid transition to technology-based models of health care delivery to ensure continuous access to health care. However, it is possible that geographic disparities in digital adoption persist and may further widen already existing healthcare disparities. Thus, to guide the effective large-scale implementation of digital tools for health care delivery, we sought to estimate geographic disparities in pre-pandemic rates of digital tool ownership and utilization [11-31].

In this study, we aimed to examine pre-pandemic rates of ownership and preferences for utilization of DHT as a measure of interest among rural adults compared to their urban counterparts in the United States using a nationally representative sample.

## Materials and methods

Population sample

In this research, we consolidated data from the Health Information National Trends Survey (HINTS), which is a household interview survey of noninstitutionalized American adults aged ≥18 years [32]. The National Cancer Institute (NCI) has periodically conducted HINTS since 2003 to evaluate trends and patterns related to demographics, perceptions and use of health information communication systems, access, and attitudes toward use of health information technology (HIT) from the general population. We utilized data from the fifth edition HINTS 5 Cycle 3 (H5c3). Data collection for Cycle 3 of HINTS 5 began in January 2019 and concluded in May 2019. Additional information about data collection and methodologies can be found in the corresponding methodology reports for HINTS 5 cycle 3 estimates [12].

Participants

Eligible information was obtained from 5,438 respondents who completed at least 50% of the survey (with 5,247 completing 80% or more). The overall household response rate was 30.3%. The H5c3 survey respondents were then weighted to reflect selection probabilities and compensate for non-response in order to provide a nationally representative sample in terms of age, sex, educational attainment, race, ethnicity, and census region.

For this study, respondents were separated into two groups: (i) rural residents group and (ii) urban residents group. Informed by prior studies [13], rural/urban residence was defined using the Rural-Urban Continuum (RUC) Code per the US Department of Agriculture Economic Research Service (2013) [14].

The dataset also contained questions about participants' ownership and usage of DHT, such as smartphones, health applications, and use of DHT to interact with the health care system. Of the complete sample, approximately 601 participants resided in rural areas. The missing data rate was 0.0% (0/5438). Written informed consent was obtained from study participants. The Westat institutional review board approved HINTS 5, Cycle 3, and it was classified exempt from review by the US National Institutes of Health Office of Human Subjects Research Protections. This study followed the Strengthening the Reporting of Observational Studies in Epidemiology (STROBE) reporting guideline.

Outcome variables

The primary measures of interest were sets of inquiries pertaining to: (a) ownership rates of DHT, (b) perceived usefulness (c) interactions with healthcare systems utilizing DHT. The questions used for analysis, along with their corresponding responses, are outlined in in the results section.

Exposure variables

The primary exposure variable in this study was self-reported residential status, which was dichotomized into two categories: rural and urban residents

Covariates

Demographic variables included were age, and gender, marital status, income, census region, health insurance and race. Other variables, including clinical and health-related factors such as self-health status, confidence in taking care of one's health, having a regular health provider, and the number of comorbidities, were also included in the models.

Statistical analyses

All statistical analyses were conducted using the “svy” command in Stata 14.0 statistical software (StataCorp LP, College Station, Texas, USA). To evaluate the ownership and use of DHT, basic descriptive statistics were conducted for the entire study sample and by urban versus rural residence status. Both unweighted frequencies and weighted percentages were also calculated. We examined the differences between rural and urban residents on ownership rates, usage, and perceived benefits of DHT, as well as utilizing digital tools to interact with health care systems by conducting unadjusted and adjusted logistic regression models. Two adjusted models were created to examine the geographical (rural versus urban) differences in HIT use to access medical information and communicate with providers. The first adjusted model (Model 1) included age, gender, race, self-rated health, household income, access to regular provider, education level, census region, confidence in taking care of self and number of comorbidities. The second model (Model 2) additionally included internet use as an added covariate, given previous studies indicating rural-urban disparities in internet use [10]. To ensure nationally representative parameter estimates, all analyses were weighted to account for the sampling design of HINTS 5 (Cycle 3). Accurate variance estimates were obtained using replicate weights based on the jackknife replication method [33]. A Bonferroni correction was applied to account for multiple comparisons, setting statistical significance at a p-value of 0.003 (0.05/14 = 0.003) [15,16].

## Results

A total of 5438 participants were included in this analysis (see Table 1). The mean age of the respondents was 55 years (standard deviation: 19.9 years). Within this sample, 601 (11.1%) participants were rural residents. At baseline, demographic characteristics were almost entirely similar for both rural and urban dwellers; however, rural residents were more likely to be white and less likely to live in a household earning $75,000 annually. All sociodemographic characteristics of both groups are reported in Table 1 below.

**Table 1:** Sample population demographic characteristics sample N = 5,438

Demographic variables	Total (n=5,438), %	Urban population (n=4,837), %	Rural population (n= 601), %	P-value
Gender				0.849
Female	50.86	50.75	51.60	
Male	49.14	49.25	48.40	
Marital Status				0.176
Not Married	44.34	45.13	39.26	
Married	55.66	55.87	60.74	
Age Group				0.366
18-34	24.27	24.53	22.61	
35-49	24.48	24.17	26.51	
50-64	31.10	31.69	27.21	
65+	20.15	19.61	23.67	
Education				0.074
Less than College	39.97	39.46	43.33	
Some college	30.56	29.87	35.04	
College graduate	17.31	17.85	13.77	
Post-graduate	12.16	12.82	7.86	
Household Income				0.011
< $20,000	18.46	17.64	24.10	
$20,000 - $34,999	11.04	10.61	13.95	
$35,000 - $49,999	13.52	13.12	16.26	
$50,000 – $74,999	17.43	17.25	18.71	
$75,000 or more	39.55	41.38	26.98	
Race				<0.001
White	63.47	61.03	79.85	
Black/African American	11.31	12.05	6.37	
Hispanic	16.82	17.81	10.23	
Others	8.40	9.11	3.55	
Insurance status				0.755
No	8.40	68.49	7.85	
Yes	91.60	91.51	92.15	
Census Region				<0.001
Northeast	17.74	19.00	9.53	
Midwest	20.88	18.75	34.90	
South	37.75	37.51	39.28	
West	23.62	24.74	16.29	
Self-Health Status				0.210
Fair or poor	15.16	14.59	18.85	
Good	35.47	35.16	37.45	
Excellent or very good	49.37	50.25	43.70	
Confidence in taking care of own health				0.387
Somewhat or none	28.45	28.01	31.33	
Completely or very	71.55	71.99	68.67	
Having a Regular Provider				0.338
No	35.51	35.05	38.50	
Yes	64.49	64.95	61.50	
Comorbidity				0.366
None	39.26	39.13	40.14	
One comorbidity	31.98	32.58	28.09	
At least two or more comorbidities	28.76	28.29	31.77	

Ownership, usage and perceived usefulness of DHT among rural vs urban residents

In both the unadjusted and adjusted models, ownership of smartphones, tablets and health apps did not differ by geographic residence. In the fully adjusted model, rural residents were as likely as urban residents to own smartphones (OR 0.81, 95% CI 0.59 - 1.11; p = 0.179), health apps (OR 0.93, 95% CI 0.62 - 1.42; p = 0.746), and tablets computers (OR 0.87, 95% CI 0.61 - 1.24; p = 0.434). However, they had lower odds of reporting internet use (OR 0.50, 95% CI 0.33 - 0.76; p = 0.002) than their urban counterparts. In addition, rural residents were as likely as those in urban populations to endorse DHT as beneficial. Both groups were equally likely to have reported their smart device as helpful in discussions with their healthcare providers (OR 0.90; 95% CI 63 - 1.30; p = 0.572). Similarly, there were similar odds of reporting that DHT had helped them to track progress on a health-related goal (e.g., quitting smoking, losing weight, or increasing physical activity) (OR 1.17; 95% CI 0.75 - 1.83; p = 0.491), and to make medical decisions (OR 1.05; 95% CI 0.70 - 1.59; p = 0.797). These relationships were statistically insignificant in the unadjusted and adjusted models (See Table 2 below). Model were adjusted for gender, race, marital status, education level, household income, health insurance status, census region, self-rated health, access to regular provider, number of comorbidities and confidence in taking care of self.

**Table 2:** Unadjusted and adjusted odds ratios (ORs) for ownership and perceived usefulness of electronic devices among Rural vs Urban Residents Model were adjusted for gender, race, marital status, education level, household income, health insurance status, census region, self-rated health, access to regular provider, number of comorbidities and confidence in taking care of self.

Question	Yes (%)		Unadjusted OR 95% C. I	p-value	Adjusted OR, 95% C. I.*	p-value
	Urban	Rural				
Please indicate if you have a smartphone.	84.10	81.64	0.84 (0.64,1.10)	0.197	0.81 (0.59, 1.11)	0.179
Please indicate if you have a tablet computer.	60.24	54.52	0.79 (0.58,1.08)	0.134	0.87 (0.61, 1.24)	0.434
On your tablet or smartphone, do you have any apps related to health and wellness?	55.35	51.41	0.85 (0.59, 1.23)	0.386	0.93 (0.62, 1.42)	0.746
Do you ever go on-line to access the Internet or World Wide Web, or to send and receive e-mail?	85.06	80.02	0.70 (0.52,0.95)	0.024	0.50 (0.33, 0.76)	0.002
In the past 12 months, have you used an electronic wearable device to monitor or track your health or activity? For example, a Fitbit, AppleWatch or Garmin Vivofit	27.38	24.15	0.84 (0.56, 1.28)	0.417	0.93 (0.60, 1.46)	0.755
Has your tablet or smartphone helped you track progress on a health-related goal, such as quitting smoking, losing weight, or increasing physical activity?	46.24	46.71	1.02 (0.71, 1.46)	0.920	1.17 (0.75, 1.83)	0.491
Has your tablet or smartphone helped you make a decision about how to treat an illness or condition?	42.38	40.51	0.93 (0.67, 1.28)	0.631	1.05 (0.70, 1.59)	0.797
Has your tablet or smartphone helped you in discussions with your health care provider?	37.69	32.13	0.78 (0.55, 1.11)	0.165	0.90 (0.63, 1.30)	0.572

Table 3 displays the unadjusted and adjusted ORs (Models 1 and 2) for the use of DHT to interact with the healthcare system among rural versus urban residents. After adjustment and correction for multiple comparisons, rural Americans were equally likely as their urban counterparts to use electronic devices to manage their health (See Table 3 below), but they were 51% less likely (OR 0.49, 95% CI 0.31 - 0.78; p = 0.003) to use electronic means to communicate with their physician’s office. However, after controlling for technology ownership (internet access; Model 2), these results were attenuated substantially, and all relationships did not meet statistical significance.

**Table 3:** Unadjusted and adjusted odds ratios (ORs) for use of DHT to interact with the healthcare system among rural vs urban residents Data collection for Cycle 3 of HINTS 5 began in January 2019 and concluded in May 2019 Model 1 was adjusted for gender, race, marital status, education level, household income, health insurance status, census region, self-rated health, access to regular provider, number of comorbidities, and confidence in taking care of self. Model 2 was adjusted for gender, race, marital status, education level, household income, health insurance status, census region, self-rated health, access to regular provider, number of comorbidities and confidence in taking care of self and internet access.

Question	Rural	Urban	Unadjusted OR	p-value	Model 1, AOR, 95% CI	p-value	Model 2, AOR, 95% CI	p-value
Have you sent a text message to or received a text message from a doctor or other health care professional within the last 12 months?	39.02	28.71	0.63 (0.47, 0.85)	0.003	0.65 (0.46, 0.91)	0.015	0.68 (0.47, 0.97)	0.036
In the past 12 months have you used a computer, smart phone, or other electronic means to buy medicine or vitamins online?	30.29	26.87	0.85 (0.59, 1.21)	0.346	0.96 (0.68, 1.35)	0.788	1.01 (0.71, 1.45)	0.943
In the past 12 months have you used a computer, smart phone, or other electronic means to look up medical test results?	41.47	28.52	0.56 (0.42, 0.75)	< .001>	0.61 (0.42, 0.88)	0.009	0.64 (0.43, 0.94)	0.024
In the past 12 months have you used a computer, smart phone, or other electronic means to make appointments with a health care provider?	45.14	28.75	0.49 (0.36, 0.68)	< .001>	0.54 (0.35, 0.84)	0.008	0.56 (0.36, 0.87)	0.012
In the past 12 months have you used a computer, smart phone, or other electronic means to look for health or medical information for yourself?	73.45	67.92	0.77 (0.58, 1.01)	0.060	0.65 (0.44, 0.97)	0.035	0.74 (0.49, 1.14)	0.171
In the past 12 months have you used a computer, smart phone, or other electronic means to use e-mail or the internet to communicate with a doctor or a doctor’s office?	45.57	28.22	0.47 (0.33, 0.67)	< .001>	0.49 (0.31, 0.78)	0.003	0.52 (0.32, 0.85)	0.010
In the past 12 months have you used a computer, smart phone, or other electronic means to track health care charges and costs?	37.30	27.19	0.63 (0.47, 0.84)	0.003	0.67 (0.46, 0.97)	0.034	0.70 (0.48, 1.03)	0.071

## Discussion

This study examined and compared DHT ownership rates, perceived usefulness, and interactions with healthcare systems using digital technology among rural versus urban residents. Our results indicate that rural Americans were as likely as their urban counterparts to own DHT (tablets, health apps, smartphones, or wearable devices). These findings herald new evidence to suggest that the geographic digital disparities may be narrowing. Importantly, our results suggest that on a national level, except for internet access, rural American appear to have comparable access to and ability to engage with other forms of technology as urban residents, indicating that these DHT may offer new opportunities to enhance access to health care in this group using technology-based interventions.

The ongoing pandemic may have shaped the use of DHT to interact with healthcare systems [17]. The percentage of individuals in rural and urban areas who have used electronic means for various healthcare activities during the pandemic has increased in the post-pandemic era [17-20]. Our findings presented two adjusted odds ratios for each healthcare activity along with their respective 95% confidence intervals and p-values. The results show that individuals in rural areas are less likely to have sent a text message to or received a text message from a doctor or other health care professional, used electronic means to look up medical test results, make appointments with a health care provider, use email or the internet to communicate with a doctor or a doctor’s office and track health care charges and costs compared to their urban counterparts. The unadjusted odds ratios for these activities are less than 1, indicating that individuals in rural areas are less likely to use electronic means for these activities.

After adjusting for other variables in Model 1, individuals in rural areas remain less likely to have sent a text message to or received a text message from a doctor or other health care professional, used electronic means to look up medical test results, make appointments with a health care provider, use email or the internet to communicate with a doctor or a doctor’s office and track health care charges and costs compared to their urban counterparts. The adjusted odds ratios for these activities are also less than 1, indicating that individuals in rural areas are still less likely to use electronic means for these activities after adjusting for other variables. After further adjusting for additional variables in Model 2, individuals in rural areas remain less likely to have sent a text message to or received a text message from a doctor or other health care professional, used electronic means to look up medical test results, make appointments with a health care provider, use email or the internet to communicate with a doctor or a doctor’s office compared to their urban counterparts. However, the adjusted odds ratios for tracking health care charges and costs and looking for health or medical information for themselves are not statistically significant in Model 2. Regarding DHT utilization, our results suggest that individuals in rural areas are less likely to use electronic means for various healthcare activities compared to their urban counterparts, even after adjusting for other variables.

Furthermore, DHT may offer promise as a tool to enhance continuity of care [2-6,17-21]. We found that rural and urban residents had similar odds of reporting having used electronic devices to look for health information, schedule appointments, track health costs, and view test results compared to urban residents. However, rural residents were less likely to utilize DHT to communicate with their physicians when compared to urban residents. Notably, this difference was attenuated after adjusting for internet access, suggesting that access to these internet services may play some role in driving the rural-urban differences in DHT use. These findings are consistent with previous research that reported disparities in internet access between rural and urban Americans [10,22,23]. This attenuation of internet service in our findings has also been reported as part of the several factors contributing to the disparities in digital health technology use between rural and urban populations [23-30]. Several other studies have also reported that high-speed internet access and usage are limited in rural areas compared to urban areas. A study conducted by the Federal Communications Commission in 2018 found that millions of Americans lacked broadband access, with the majority of them living in rural areas [22-26]. This lack of internet access limits the use of telehealth services and remote patient monitoring, which are critical components of digital health technology.

Our results also showed fewer college and post-college graduates in the rural population compared to the urban population as a potential correlate to the disparity in DHT. This may have justified the ownership and perceived perspective on internet surfing or the use of emails (p-value = 0.024). Some studies have shown a lack of awareness and education about digital health technology in rural areas that are less educated. A United States Department of Health and Human Services study reported that higher education rates were higher in urban areas than in their rural counterpart (HHS) [20]. This may translate to lower education and utilization of DHT in rural settings. Our findings have also aligned the perspective with some studies that have also shown a lack of awareness and education about digital health technology in rural areas that have less educated residents [21-25]. A study conducted by Pew Research Center in 2019 found that only 63% of rural Americans have home broadband, compared to 75% of urban Americans [24-28]. The lack of access to information and educational resources limits the ability of rural residents to adopt and utilize digital health technologies effectively.

Our result also showed that income disparity between rural and urban dwellers may have also played a role. There was a cost barrier to digital health technology adoption and utilization in rural areas (p= 0.024 and 0.012) for usage to look up medical devices and schedule appointments within the last 12 months. According to National Rural Health Association studies, many rural residents are uninsured or underinsured, and the cost of digital health technology may be prohibitive. According to a National Rural Health Association report, 20% of rural residents are uninsured, compared to 12% of urban residents [29]. Other studies have identified several factors like health illiteracy, sociodemographic factors, and the structural system as barriers to health information technology adoption in rural areas [20-22]. Our results further extend the literature and confirm internet access is a crucial factor that may contribute to the low rates of DHT adoption observed in rural populations.

Implications

Rural residents own digital tools at comparable rates to the general population offering a potential tool to improve access to healthcare in such areas. However, disparities in internet access continue to exist and may be the major challenge to the widespread implementation of DHT in rural areas. Policymakers, healthcare providers, and organizations in rural areas can integrate digital tools such as “low connectivity” apps that require minimal internet access temporarily in delivering healthcare to rural Americans till digital infrastructure catches up. Given the lack of internet access in these rural areas, funding should be increased to create more broadband services in these areas. The Rural Healthcare Program, an initiative from the Federal Communications Commission (FCC) to provide funding to eligible healthcare providers in rural communities, is capped at $400 million annually, which has not increased since 1997 [22,23,26-30]. Since the rural population and access to DHT continue to increase, this calls for review and possible enhancement in funding for broadband access.

Limitations

Despite our results, some limitations exist. Firstly, the cross-sectional nature of the study design precludes any ability to draw causal inferences. Secondly, the variables were self-reported, introducing the likelihood of recall bias. Third, the response rate for HINTS 2019 data was 30.3%, which raises the potential for selection bias. Fourth, it is important to highlight that our analysis focused on pre-pandemic rates of DHT ownership and utilization. It is possible that rates of DHT ownership and use may have increased during the COVID-19 pandemic. Lastly, the dataset did not include several possible unmeasured confounders that may explain the observed relationships, such as health literacy.

## Conclusions

DHT has emerged as an effective tool to augment health care delivery. We found that rural residents are equally likely as urban residents to own and use DHT to manage their health. However, they were less likely to communicate with their health providers using DHT. Our results suggest that individuals in rural areas are less likely to use electronic means for various healthcare activities than their urban counterparts, even after adjusting for other variables. Furthermore, our results suggest that disparities in internet access may be driving these disparities. Addressing these disparities will require a multifaceted approach that addresses the lack of access to high-speed internet, the shortage of healthcare providers, the lack of awareness and education, and the cost barrier to adoption. Therefore, better health policies or research aimed at reducing these DHT gaps and reasons and strategies to eliminate barriers to more digital adoption in rural communities are crucially needed.

## References

[1] Blumenthal D, Abrams M, Nuzum R (2015). The affordable care act at 5 years. N Engl J Med.

[2] Cyr ME, Etchin AG, Guthrie BJ, Benneyan JC (2019). Access to specialty healthcare in urban versus rural US populations: a systematic literature review. BMC Health Serv Res.

[3] Blumenthal SJ, Kagen J (2002). The effects of socioeconomic status on health in rural and urban America. JAMA.

[4] (2023). Rural health. https://www.cdc.gov/ruralhealth/index.html.

[5] Schueller SM, Hunter JF, Figueroa C, Aguilera A (2019). Use of digital mental health for marginalized and underserved populations. Current Treatment Options in Psychiatry.

[6] Kulcsar Z, Albert D, Ercolano E, Mecchella JN (2016). Telerheumatology: a technology appropriate for virtually all. Semin Arthritis Rheum.

[7] Mohr NM, Vakkalanka JP, Harland KK, Bell A, Skow B, Shane DM, Ward MM (2018). Telemedicine use decreases rural emergency department length of stay for transferred North Dakota trauma patients. Telemed J E Health.

[8] Southard EP, Neufeld JD, Laws S (2014). Telemental health evaluations enhance access and efficiency in a critical access hospital emergency department. Telemed J E Health.

[9] Bhuyan SS, Lu N, Chandak A (2016). Use of mobile health applications for health-seeking behavior among US adults. J Med Syst.

[10] Greenberg AJ, Haney D, Blake KD, Moser RP, Hesse BW (2018). Differences in access to and use of electronic personal health information between rural and urban residents in the United States. J Rural Health.

[11] Wang JY, Bennett K, Probst J (2011). Subdividing the digital divide: differences in internet access and use among rural residents with medical limitations. J Med Internet Res.

[12] NCI. (2019, August August (2019). Health Information National Trends Survey 5 (HINTS 5) Cycle 3 Methodology Report. Health Information National Trends Survey 5 (HINTS 5) Cycle 3 Methodology Report. https://hints.cancer.gov/data/methodology-reports.aspx.

[13] Zahnd WE, Davis MM, Rotter JS (2019). Rural-urban differences in financial burden among cancer survivors: an analysis of a nationally representative survey. Support Care Cancer.

[14] (2023). Rural-Urban Continuum Codes. http://www.ers.usda.gov/data-products/rural-urban-continuum-codes.aspx.

[15] Rust KF, Rao JN (1996). Variance estimation for complex surveys using replication techniques. Stat Methods Med Res.

[16] Curtin F, Schulz P (1998). Multiple correlations and Bonferroni's correction. Biol Psychiatry.

[17] Olatunde H, Okobi OE (2021). Telepsychiatry or face-to-face visit, the near paradigm shift in psychiatry. International Journal of Scientific Advances.

[18] Onyeaka HK, Zambrano J, Longley RM, Celano CM, Naslund JA, Amonoo HL (2021). Use of digital health tools for health promotion in cancer survivors. Psychooncology.

[19] Carswell K, Harper-Shehadeh M, Watts S (2018). Step-by-Step: a new WHO digital mental health intervention for depression. Mhealth.

[20] Chen X, Orom H, Hay JL, Waters EA, Schofield E, Li Y, Kiviniemi MT (2019). Differences in rural and urban health information access and use. J Rural Health.

[21] Krakow M, Hesse BW, Oh A, Patel V, Vanderpool RC, Jacobsen PB (2019). Addressing rural geographic disparities through health it: initial findings from the Health Information National Trends Survey. Med Care.

[22] (2023). 2018 Broadband Deployment Report. https://www.fcc.gov/reports-research/reports/broadband-progress-reports/2018-broadband-deployment-report.

[23] FCC. (2018, June 25 (2023). Federal Communications commission. https://docs.fcc.gov/public/attachments/FCC-18-82A1.pdf.

[24] (2023). US Department of Health and Human Services. Rural Health Disparities Report. https://www.ahrq.gov/sites/default/files/wysiwyg/research/findings/nhqrdr/2021qdr-appendixes.pdf.

[25] Haggstrom DA, Lee JL, Dickinson SL (2019). Rural and urban differences in the adoption of new health information and medical technologies. J Rural Health.

[26] Shatzkes Shatzkes, M. (2021, January 26 (2023). FCC Initial Connected Care Pilot Program Projects For Telehealth. https://www.natlawreview.com/article/fcc-announces-initial-connected-care-pilot-program-projects-telehealth.

[27] (2023). US Economic Resaerch services; Rural Education. https://www.ers.usda.gov/topics/rural-economy-population/employment-education/rural-education.

[28] (2021). Some digital divides persist between rural, urban and suburban America. https://www.pewresearch.org/fact-tank/2021/08/19/some-digital-divides-persist-between-rural-urban-and-suburban-america/.

[29] (2023). About Rural Health Care. https://www.ruralhealth.us/about-nrha/about-rural-health-care.

[30] Zahnd WE, Goldfarb J, Scaife SL, Francis ML (2010). Rural-urban differences in behaviors to prevent skin cancer: an analysis of the Health Information National Trends Survey. J Am Acad Dermatol.

[31] Whitacre BE, Williams RS (2015). Electronic medical record adoption in Oklahoma practices: rural-urban differences and the role of broadband availability. J Rural Health.

[32] (2023). What is HINTS?. https://hints.cancer.gov/.

[33] Lumley T (2004). Analysis of complex survey samples. Journal of Statistical Software.

